# Influência de um Protocolo de Mobilização Precoce no comportamento autonômico de pacientes submetidos a Angioplastia Coronária Transluminal Percutânea

**DOI:** 10.36660/abc.20200296

**Published:** 2021-11-22

**Authors:** Bárbara Oliveira Silveira, Jade Lara de Melo, Graziella Paula de Oliveira Neri, Michele Lima Gregório, Moacir Fernandes de Godoy, Marilita Falangola Accioly

**Affiliations:** 1 Universidade Federal do Triângulo Mineiro Uberaba MG Brasil Universidade Federal do Triângulo Mineiro, Uberaba, MG – Brasil; 2 Faculdade de Medicina de São José do Rio Preto São Jose do Rio Preto SP Brasil Faculdade de Medicina de São José do Rio Preto (FAMERP) - Pós Graduação Enfermagem, São Jose do Rio Preto, SP – Brasil; 3 Faculdade de Medicina de São José do Rio Preto São Jose do Rio Preto SP Brasil Faculdade de Medicina de São José do Rio Preto (FAMERP) - Cardiologia e Cirurgia Cardiovascular, São Jose do Rio Preto, SP – Brasil

**Keywords:** Doenças Cardiovasculares/mortalidade, Infarto do Miocárdio, Deambulação Precoce/métodos, Exercícios, Sistema Nervoso Autônomo, Angioplastia

## Abstract

**Fundamento:**

Gráficos de recorrência (GR) permitem uma análise não linear da variabilidade de frequência cardíaca (VFC) e fornecem informações sobre o sistema nervoso autônomo (SNA).

**Objetivos:**

Avaliar se a mobilização precoce em pacientes submetidos a angioplastia coronária transluminal percutânea (ACTP) influencia os componentes quantitativos e qualitativos dos GR.

**Métodos:**

Um total de 32 participantes que foram submetidos a ACTP foram divididos entre um grupo de controle (GC - sem exercícios físicos) e grupo de mobilização precoce (GMP - com exercícios físicos) A frequência cardíaca batimento a batimento foi registrada utilizando um cardiofrequencímetro em ambos os grupos na admissão e na alta. Os índices lineares nos domínios de tempo e frequência foram analisados, bem como os índices não lineares obtidos pelos GR. O protocolo fisioterapêutico de mobilização precoce começou 12-18 horas após a ACTP. Um teste T não pareado bicaudal foi utilizado para as comparações, e p-valores <0,05 foram aceitos como significativos.

**Resultados:**

Ao comparar os dois grupos, na alta, o GMP apresentou um aumento no SDNN (23,55 ± 12,05 a 37,29 ± 16,25; p=0,042), índice triangular (8,99 ± 3,03 a 9,66 ± 3,07; p=0,014) e VLF (694,20 ± 468,20 a 848,37 ± 526,51; p=0,004), mas não apresentou alterações significativas na avaliação não linear. Além disso, na análise qualitativa dos GR, observou-se um padrão mais difuso e menos geométrico no GMP, indicando maior variabilidade, enquanto no GC, notou-se um padrão geométrico mais alterado.

**Conclusão:**

O protocolo de mobilização precoce promove uma melhoria no comportamento autonômico, conforme avaliado por VFC e GR, e pode ser considerado um procedimento útil para a melhor recuperação de pacientes submetidos a ACTP.

## Introdução

As doenças cardiovasculares são responsáveis pelo número mais alto de mortes em todo o mundo.¹ Entre elas, o infarto do miocárdio agudo (IMA) é a principal causa de mortalidade no Brasil.² Entretanto, a sobrevida desses pacientes aumentou devido a avanços tecnológicos, tais como a angioplastia coronária transluminal percutânea (ACTP).^3,4^

Em associação com a ACTP, a interação multidisciplinar desempenha um papel importante na redução da mortalidade,^5^ uma vez que a mobilização precoce evita o confinamento ao leito e seus vários efeitos deletérios, tais como o declínio funcional e a redução da qualidade de vida após a alta.^6^ Entretanto, ainda é muito comum que pacientes permaneçam confinados ao leito por receio de instabilidade hemodinâmica.^7^

Em contraste, a análise da modulação autonômica cardíaca pela variabilidade de frequência cardíaca (VFC) foi utilizada como preditor de riscos cardiovasculares em várias condições.^8,9^ Entretanto, a maioria dos estudos utilizou a análise linear da VFC.^10-12^

O corpo humano é uma boa ilustração de um “sistema complexo” caracterizado pela interação contínua de seus vários órgãos para manter a vida. Sua complexidade resulta em um modo de comportamento que é tipicamente não linear em situações normais.^13^ Assim, os métodos relacionados à dinâmica não linear são geralmente mais relevantes clinicamente para uma melhor interpretação do comportamento fisiopatológico da VFC sob várias condições, e, consequentemente, seu valor prognóstico, complementando as informações obtidas com avaliações tradicionais.^14^

Gráficos de recorrência (GR) são um método de análise não linear idealizado por Eckmann et al.,^15^ que propõe a análise do comportamento de um sistema, representado por uma série temporal, em um espaço abstrato chamado espaço fásico, permitindo a quantificação e a qualificação da VFC.^15,16^

Além disso, pouco se sabe sobre respostas agudas ao exercício precoce na modulação autonômica e função cardiovascular no período pós-operatório imediato de pacientes submetidos a revascularização do miocárdio e ACTP.^17^

Portanto, o presente estudo pretendeu avaliar se a mobilização precoce em pacientes submetidos a ACTP influencia os componentes quantitativos e qualitativos dos GR.

## Métodos

### Amostra

Esse é um ensaio clínico prospectivo, controlado e quase-experimental. A amostra incluiu indivíduos que foram submetidos a ACTP no Hospital de Clínicas da Universidade Federal do Triângulo Mineiro.

O tamanho da amostra foi calculado considerando-se a prevalência de indivíduos que precisam de angioplastia e estão hospitalizados em uma unidade de cuidado coronário. Para o cálculo, foi utilizada a seguinte fórmula: n= Z^2^ x p (p-100) / e^2^, em que “Z” é o valor crítico constante que corresponde ao intervalo de confiança de 95% (IC95%); “p” é a prevalência da doença/principal variável; e “e” é o erro de amostragem, que pode variar em até 10% do valor real da população selecionada para a amostra, sugerindo uma amostra de 15 indivíduos para cada grupo.

**Figura 1:**
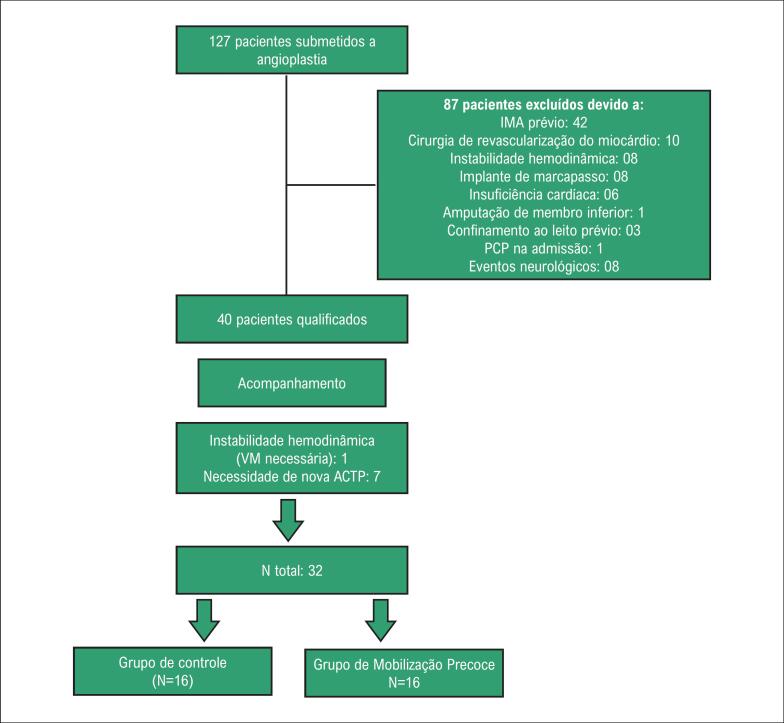
Fluxograma mostrando o recrutamento e a seleção dos participantes do estudo. IMA: Infarto do miocárdio agudo. PCP: Parada cardiopulmonar ACTP: Angioplastia coronária transluminal percutânea. VM: Ventilação mecânica.

O fluxograma para recrutamento e seleção de amostra é apresentado na Figura 1.

A amostra foi composta de 32 participantes que atenderam aos seguintes critérios de inclusão: ter pelo menos 18 anos de idade; apresentar um diagnóstico médico de um IMA sem complicações (Killip I ou II), com ou sem supradesnivelamento do segmento ST e/ou indicação de ACTP eletiva (ACTP bem-sucedida com estenose residual de menos de 50%); ser capaz de entender as instruções para realizar exercícios físicos. Em seguida, os participantes foram divididos em dois grupos: Grupo de mobilização precoce (GMP) com 16 participantes submetidos ao protocolo de mobilização precoce; e grupo de controle (GC) com 16 participantes não submetidos ao protocolo de mobilização precoce. A amostra foi ajustada para idade, sexo e diagnóstico médico.

Indivíduos com pelo menos uma das características abaixo foram excluídos do estudo: histórico prévio de IMA, IMA com complicações (Killip III e IV), implante de marcapasso, bloqueio atrioventricular de 2º ou 3º grau, sequela de acidente vascular cerebral, amputação de membro inferior, estenose aórtica grave, cirurgia de revascularização do miocárdio prévio, insuficiência cardíaca, instabilidade hemodinâmica em repouso, piora da condição clínica geral, condição febril, insuficiência respiratória (necessidade de ventilação mecânica).

Os procedimentos do estudo seguiram todas as normas da Resolução nº 466 do CNS e foi aprovado pelo Comitê de Ética e Pesquisa da Universidade Federal do Triângulo Mineiro sob o número de resolução 2.319.890 e pelo Registro Brasileiro de Ensaios Clínicos sob o número RBR-9w378x.

### Protocolo experimental

O protocolo experimental consistiu em quatro fases. A fase I foi conduzida com entrevistas e avaliação de registros médicos. A fase II foi realizada de 12 a 18 horas após a ACTP e consistiu em registrar a frequência cardíaca batimento a batimento, utilizando um cardiofrequencímetro Polar® modelo RS800CX (Polar Kempele, Finlândia), e coletando o tacograma continuamente por 20 minutos. Durante todo o processo de coleta, o participante permaneceu em repouso, em posição supina, em silêncio e acordado. A fase III foi caracterizada pela implementação de um protocolo de mobilização precoce, realizado apenas pelo GMP.

Na fase IV, foi realizado um novo eletrocardiograma para a analisar a VFC, seguindo o mesmo procedimento realizado na fase II.

### Protocolo de Mobilização Precoce

O Protocolo de Mobilização Precoce foi adaptado do protocolo utilizado no Grady Memorial Hospital e na Escola de Medicina da Emory University^18^ composto de passos progressivos em posições diferentes, conforme descrito na Tabela 1. O protocolo foi iniciado e aplicado nas posições de acordo com o status funcional do participante, que foi verificado por avaliações e comunicação com a equipe multidisciplinar. O GMP realizou o protocolo durante o período de hospitalização, que consistiu em duas sessões por dia (4 intervenções durante a permanência da UTI).

**Tabela 1:** Protocolo de Mobilização Precoce

Posição	Exercícios
Supina	A) Um set de exercícios de diafragma com movimento diagonal ativo-assistido ou ativo-livre do membro superior (10 vezes para cada membro);
	B) Repetir o exercício por um set com ambos os membros simultaneamente (10 vezes);
	C) Repetir o exercício por um set com ambos os membros simultaneamente (10 vezes);
	D) Flexão tripla (10 vezes) + abdução/adução do quadril ativas-assistidas ou ativas-livres (10 vezes para cada membro);
	E) Flexão-extensão de tornozelo (10 vezes) para cada pé;
	E) Circundução de tornozelo (10 vezes) para cada pé;
Sentado	Exercícios ativos de A a F
Ortostatismo	Exercícios ativos de A a F e ativos para membros inferiores (suportado, se necessário)
	G) Ficar nas pontas dos pés (10 vezes) com ambos os membros simultaneamente;
	H) Meio agachamento (10 vezes);
	I) Marcha no lugar (por 30 segundos);
	J) Deambulação no quarto (1 volta).

Os seguintes critérios foram utilizados para interromper o Protocolo de Mobilização Precoce: sinais e/ou sintomas de fadiga, dor no peito, dispneia, cianose, palidez, taquicardia (>120 batimentos por minuto), bradicardia, arritmias complexas e hipotensão (Pressão arterial média <65mmHg).

### Avaliação de modulação autonômica

Para a análise de índices de VFC, registros de intervalo de RR foram transmitidos para um computador utilizando o software Polar Precision Performance (versão 4.01.029)^19^ e convertidos em arquivo de texto. Apenas as séries com mais de 95% de batimentos sinusais foram analisadas, após a seleção dos 1000 pontos mais estáveis (Kubios HRV Software, versão 2.0, Universidade de Kuopio, Finlândia). Os dados foram filtrados usando o filtro padrão do software Polar Precision Performance (versão 4.01.029), com filtro moderado. Em seguida, uma ferramenta de filtragem computacional chamada T-RR Filter^20^ foi utilizada.

Entre os métodos lineares, o domínio tempo mede os intervalos RR normais (iR-R) e as várias medições são calculadas a partir desses intervalos, incluindo: desvio padrão do iR-R normal médio (SDNN), que corresponde a efeitos simpáticos e parassimpáticos, e representam variabilidade global; a porcentagem do iR-R adjacentes com diferença de duração acima de 50 milissegundos (pNN50); e a raiz quadrada média das diferenças sucessivas entre os iR-R adjacentes usuais (RMSSD). As variáveis RMSSD e pNN50 estão relacionadas apenas ao comportamento parassimpático, enquanto o SDNN reflete todos os componentes responsáveis pela variabilidade. O índice triangular refere-se ao número de todos os iR-R dividido pela frequência desses iR-R no compartimento modal do histograma, refletindo, dessa forma, a variabilidade global.^21^

Para a análise do domínio frequência, o método de interpolação por spline cúbica em 4Hz foi aplicado, e a densidade espectral de potência do segmento mais estável foi calculada utilizando-se a Transformação rápida de Fourier (FFT), calculando, em milissegundos ao quadrado (ms^2^), os componentes espectrais para banda de frequência muito baixa (VLF) (<0,04Hz), frequência baixa (LF) (0,04-0,15 Hz), e frequência alta (HF) (0,15 a 0,40 Hz), além da relação entre esses componentes (LF/HF).

LF representa a modulação parassimpática; HF representa a atividade de modulação vagal.^22^ VLF reflete regulações humorais, vasculares, térmicas, bem como a atividade do sistema renina-angiotensina-aldosterona.^22^ Além disso, a relação entre LF e HF pode ser uma medida de balanço simpatovagal.^22,23^

Os GR podem ser usados para análise não linear de VFC, que foi realizada de maneira qualitativa e quantitativa. A análise qualitativa foi realizada pela visualização do padrão gráfico e a análise quantitativa utilizou os seguintes índices: Recorrência (REC), Determinismo (DET), Entropia de Shannon (ES), Laminaridade (LAM), Tempo de permanência (TT) e Comprimento máximo de linha (MaxLine).^15^ Os seguintes parâmetros foram usados nos GR: dimensão de incorporação= 10; atraso= 1, raio= 70, linha= 2^24^ e esquema de cores= Volcano.

É utilizada uma série temporal para a construção do GR. De acordo com faixas de valores entre medidas (dimensões) e distâncias ou intervalos de tempo (raio), pode-se verificar se existem valores de recorrência ou não. O uso de cores diferentes representa vários raios, complementando a aparência visual típica dos GR.^24^

Os GR podem ser analisados por padrões de pequena ou grande escala. Os padrões de pequena escala se encaixam em pontos, e linhas diagonais e verticais, o que permite uma análise qualitativa. Por exemplo, em indivíduos saudáveis, o GR mostra uma linha diagonal e menos quadrados aparentes, indicando uma VFC mais alta. Em indivíduos com alguma deficiência na modulação autonômica, o GR mostra quadrados definidos no gráfico, mais formas geométricas, indicando a periodicidade inerente, comportamento linear e VFC baixa.^24,25^

Na análise quantitativa, alguns índices são gerados: índice de recorrência (REC%), que quantifica a porcentagem de pontos recorrentes com um raio específico; porcentagem de determinismo (DET%), representando as linhas diagonais formadas pelos pontos de recorrência; comprimento médio das linhas diagonais (Lmean) e comprimento máximo das linhas diagonais (Lmax), representando a maior diagonal exceto a principal; laminaridade (LAM), que são os pontos de recorrência que formam linhas verticais; tempo de permanência (TT), que é o comprimento médio das linhas verticais; entropia, representando a entropia de Shannon, que mede a complexidade de distribuição de linhas diagonais. Nesse caso, diferentemente de outras interpretações, entende-se que quanto mais alta a entropia de Shannon, mais linear será a série temporal.^25^

**Figura 2:**
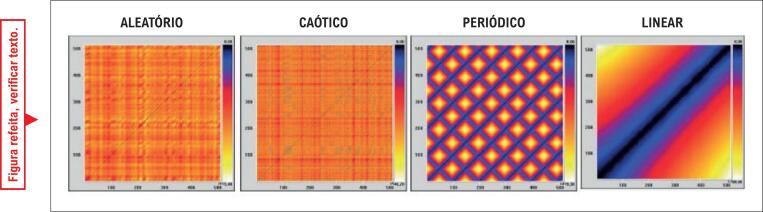
GR de sistemas aleatórios, caóticos, periódicos e lineares obtidos com fórmulas matemáticas.

Para a análise qualitativa, sistemas caóticos, aleatórios, periódicos e lineares construídos por meio da fórmula matemática relatada por Takakura et. al.^24^ foram usados como modelos de GR, conforme mostrado na Figura 2.

Fonte: Takakura et al.,^21^

### Análise estatística

Foi realizada a análise descritiva de variáveis categóricas e contínuas. A presença ou ausência de distribuição normal de variáveis foi avaliada por testes de Shapiro-Wilk. Dados contínuos com distribuição gaussiana foram expressos como média ± desvio padrão. Variáveis categóricas foram expressas como valores absolutos e porcentagens.

Um teste T não pareado bicaudal foi definido para análises intergrupos, admitindo-se um erro alfa de 5%. Todas as análises foram realizadas com o software Stats Direct Statistical versão 3.3.3.

## Resultados

A amostra analisada foi homogênea em relação a diagnóstico clínico, pressão arterial sistêmica, frequência cardíaca em repouso, duração da internação, número de stents implantados, e índices de VFC. A tabela 2 descreve a caraterização das amostras de ambos os grupos.

**Tabela 2:** Caracterização de amostras

	Grupo de controle (N=16)	Grupo de Mobilização Precoce (N=16)	p-valor*
**Perfil sociodemográfico**			
Idade: anos	61,00 ± 8,34	63,50 ± 9,64	0,439
Sexo: masculino (n)	08 (50%)	08 (50%)	
IMC (kg/m²)	27 ± 3	29 ± 3	0,467
**Diagnóstico**			
Doença Arterial Coronariana (n)	08 (50%)	08 (50%)	
Infarto do Miocárdio Agudo (n)	08 (50%)	08 (50%)	
**PA/FC (em repouso)**			
Frequência Cardíaca (bpm)	71,38 ± 11,09	71,19 ± 10,08	0,960
Pressão arterial sistólica (mmHg)	130,25 ± 22,62	134,38 ± 19,26	0,583
Pressão arterial diastólica (mmHg)	76,50 ± 17,29	72,06 ± 9,95	0,383
Pressão arterial média (mmHg)	94,06 ± 21,22	92,69 ± 13,05	0,827
**Obstrução coronária**			
Artéria coronária direita (n)	12 (75%)	10 (62,5%)	
Anterior descendente (n)	8 (50%)	8 (50%)	
Circunflexa (n)	2 (12,5%)	3 (18,8%)	
**Duração da internação (dias)**	3,94 ± 1,48	3,75 ± 1,18	0,113
**Número de stents**	1,63 ± 0,71	1,94 ± 0,85	0,215

N: número de participantes; média ± desvio padrão; IMC: índice de massa corporal; kg/m2; quilograma por metro quadrado; PA: pressão arterial; FC: frequência cardíaca; bpm: batimentos por minuto; mmHg: milímetros de mercúrio

* teste t de Student não pareado.

Os medicamentos usados pelo GC foram betabloqueadores (81,3%), hipolipemiantes (62,5%), IECA (50%), Aspirina (62,5%) e Ticlopidina/clopidogrel (18,8); enquanto o GMP utilizou (87,5%), hipolipemiantes (53,6%), IECA (50%), Aspirina (62,5%) e Ticlopidina/clopidogrel (37,5%).

A tabela 3 resume a análise de índices lineares pelos domínios de tempo e frequência, em que se pode observar uma diferença estatisticamente significativa no SDNN, índice triangular e VLF, ao comparar GC e GMP. A Tabela 4 mostra a análise quantitativa de índices não lineares pelo software Visual Recurrence Analysis. Não há diferenças estatísticas entre ambos os momentos, ao comparar GC e GMP.

**Tabela 3:** Variabilidade de Frequência Cardíaca analisada por Métodos Lineares: Controle vs. Mobilização Precoce

Índices de VFC	Controle		Mobilização Precoce		Controle x Mobilização Precoce	
	Admissão	Alta	Admissão	Alta	Admissão	Alta
	Média (DP)	Média (DP)	Média (DP)	Média (DP)	p-valor	p-valor
**Domínio tempo**						
RR média	894,58 ±112,65	825,29 ±85,04	890,60 ±147,14	908,12 ±140,25	0,9321	0,0544
SDNN	27,73 ±18,91	27,4 ±8,71	23,55 ±12,05	37,29 ±16,25	0,4610	0,0428*
RMSSD	21,98 ±11,10	15,69 ±9,09	19,68 ±11,03	20,48 ±13,47	0,5601	0,2482
PNN50	4,19 ±7,65	3,83 ±6,85	2,30 ±5,26	5,12 ±6,53	0,7046	0,4864
Índice triangular	9,34 ±4,99	7,23 ±2,09	8,99 ±3,03	9,66 ±3,07	0,8126	0.0148*
TINN	142,81 ± 54,25	131,25 ±39,39	120,62 ±63,03	130,0 ±70,75	0,2944	0,9513
**Domínio Frequência**						
Potência de VLF (ms²)	927,23 ±1395,20	407,56 ±237,61	694,20 ±468,20	848,37 ±526,51	0,5308	0,0043*
Potência de LF (ms²)	300,06 ±367,49	177,81 ±154,94	233,93 ±165,23	380,50 ±513,86	0,5165	0,1414
Potência de HF (ms²)	148,43 ±174,09	159,87 ±223,07	94,12 ±117,58	177,68 ±159,76	0,4677	0,3282
Potência de LF/HF (ms²)	2,86 ±2,12	3,39 ±2,49	2,80 ±1,61	3,94 ±3,65	0,9295	0,6205

RR média: média de intervalos RR; RMSSD: raiz quadrada média das diferenças sucessivas entre os iR-R adjacentes usuais; pNN50: porcentagem do iR-R adjacentes com diferença de duração acima de 50 milissegundos; SDNN: desvio padrão da média de intervalos RR; índice tri RR: índice triangular de RR; TINN: Interpolação triangular de histograma de intervalos NN. Dados expressos como média (desvio padrão); VLF: Frequência Muito Baixa; HF: Alta Frequência; LF: Baixa Frequência; LF/HF: Relação entre Baixa e Alta Frequência

* diferença estatisticamente significativa (p <0,05). Teste T de Student não pareado (Controle x Mobilização Precoce).

**Tabela 4:** Variabilidade de Frequência Cardíaca analisada por Métodos Não Lineares: Controle x Mobilização Precoce

Índices de VFC	Controle		Mobilização Precoce		Controle x Mobilização Precoce	
	Admissão	Alta	Admissão	Alta	Admissão	Alta
	Média (DP)	Média (DP)	Média (DP)	Média (DP)	p-valor	p-valor
**Visual Recurrence Analysis (VRA)**						
Média	890,64 ± 147,15	908,22 ± 140,36	893,71 ± 101,13	843,18 ± 90,72	0,9457	0,1318
Porcentagem de recorrência	40,71 ±7,56	40,18 ±2,41	38,54 ±3,15	43,60 ± 13,66	0,3021	0,3389
Porcentagem de determinismo	86,35 ±10,99	86,65 ±11,37	83,71 ±13,78	86,63 ±11,39	0,553	0,8099
Porcentagem de laminaridade	92,38 ±6,29	91,05 ±9,61	89,62 ±9,66	91,51 ±9,77	0,3477	0,8941
Tempo de permanência	11,65 ±12,15	8,73 ±3,11	7,68 ±3,67	7,82 ±2,40	0,2271	0,3668
Relação	2,14 ±0,23	2,12 ±0,22	2,16 ±0,25	2,14 ±0,28	0,8472	0,8257
Entropia	3,79 ±0,75	3,83 ±0,52	3,66 ±0,60	3,71 ±0,48	0,5886	0,5117
MaxLine	219,93 ± 191,23	241,68 ± 223,29	238,12 ± 166,34	171,12 ± 95,56	0,7761	0,2587

Dados expressos como média (desvio padrão)*: diferença estatisticamente significativa (p <0,05). Teste T pareado (amostras paramétricas) e teste de Wilcoxon (amostra não paramétrica). teste T de Student não pareado (Controle x Mobilização Precoce).

**Figura 3:**
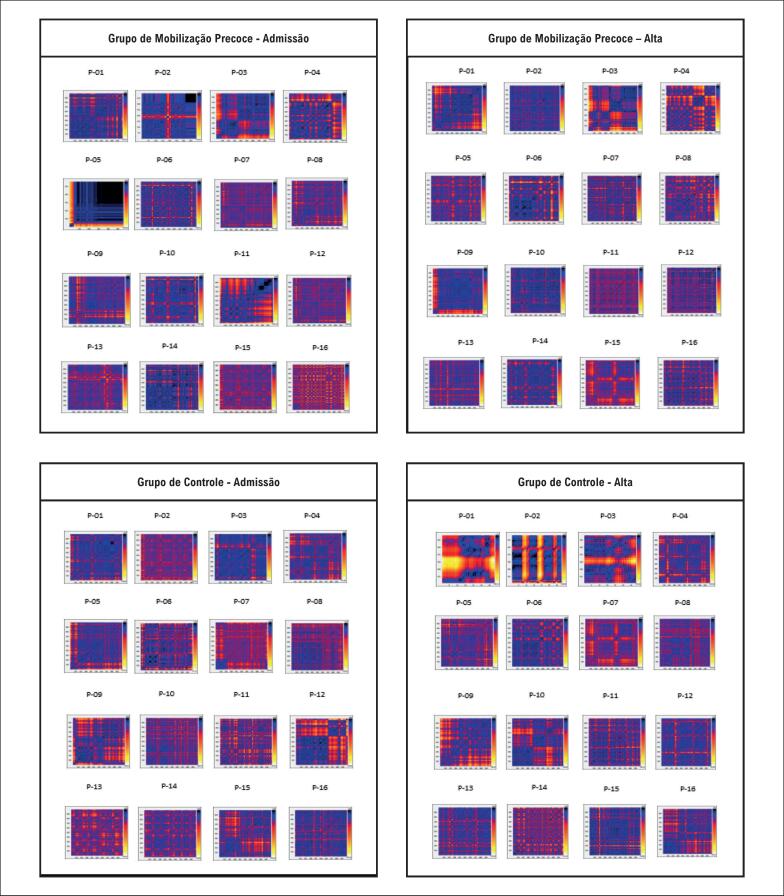
Gráficos de recorrência de participantes identificado por P_00 admissão e alta.

Entretanto, uma piora geral do padrão do Grupo de controle pode ser observada na Figura 3, que mostra a análise qualitativa do GR. Na alta, há um número maior de quadrados escuros e mais formas geométricas, indicando linearidade mais alta, e, portanto, menos homeostase do sistema, quando comparado com a admissão. Além disso, ao comparar GMP e GC, observa-se um padrão caótico, com menos quadrados aparentes e mais homogêneos no GMP, indicando maior complexidade e melhoria da homeostase.

Fonte: Pesquisa.

## Discussão

Os principais achados da análise intragrupo do GMP foram o aumento significativo dos índices lineares (SDNN e pNN50) na alta, quando comparados aos da admissão. Na análise intergrupo do GMP, observa-se um padrão mais complexo e caótico.

O aumento de índices lineares é indicativo de VFC alta, representada pelo aumento da atividade parassimpática em pNN50, e atividade autonômica global em SDNN, após a aplicação do Protocolo de Mobilização Precoce. Esse resultado implica a melhoria da saúde global do sistema cardiovascular, considerando que o aumento da atividade parassimpática está relacionado à VFC mais alta, e à mortalidade cardiovascular mais baixa,^26,27^ demonstrando a influência benéfica dos exercícios realizados na UTI.

Na análise intergrupo, os índices de SDNN, Índice triangular, e VLF demonstram uma diferença estatisticamente significativa na alta. Os índices de SDNN implicam as informações globais na modulação simpática e parassimpática; o Índice triangular expressa variabilidade no intervalo de RR, intimamente relacionada a SDNN; e a VLF está relacionada ao sistema angiotensina-aldosterona renina, termorregulação, e tônus vasomotor periférico.^25^

Alguns estudos^27,28^ demonstraram evidência de que a mobilização precoce aumenta a modulação autonômica em indivíduos após o IMA. Entretanto, esses estudos utilizaram métodos lineares em suas análises, e estudos recentes indicam que o corpo humano tem um comportamento não linear. Portanto, a análise da modulação autonômica por métodos não lineares é crucial.^14^

A análise não linear, diferentemente da análise linear, mede qualidade, escala e correlação de propriedades de sinais, proporcionando, dessa forma, uma interpretação de imprevisibilidade, complexidade e fractalidade do sistema.^14^ Meyerfeldt et al.,^26^ identificaram “sensibilidade” mais alta de análise não linear comparada à análise em comportamentos patológicos, tais como as taquiarritmias.

Ao analisar os resultados de índices para domínios de caos, em valores absolutos, não foram encontradas diferenças estatisticamente significativas. Entretanto, ao analisar o aspecto qualitativo do GR (Figura 3), é possível observar que o GR dos participantes tratados com mobilização precoce apresenta melhoria da VFC, já que apresentam cores mais heterogêneas e menos padrões geométricas. Comparados a modelos matemáticos (Figura 2), eles apresentam um comportamento que tendem ao caos, indicando maior variabilidade e, portanto, melhoria autonômica. O estudo realizado por Manata et al.^30^ corroboram os achados deste estudo, que utilizaram GR para comparar indivíduos saudáveis a indivíduos com doença pulmonar obstrutiva crônica (DPOC). Os autores concluíram que indivíduos portadores de DPOC têm VFC mais baixa, já que o GR apresentou quadrados mais visíveis e uma configuração mais homogênea. Também se observaram pontos de recorrência mais altos na comparação com indivíduos saudáveis, o que indica um sistema mais recorrente e menos dinâmico, e menos modulação autonômica complexa nessa população.

Godoy & Gregório^31^ ao analisar o GR de vários grupos, conseguiram verificar uma diferença na variabilidade desses indivíduos (recém-nascidos, adultos, portadores de doenças renais, e indivíduos com morte cerebral declarada), para as análises quantitativas e qualitativas de GR. A evidência parece corroborar nossos achados, já que foi observada a influência positiva da mobilização precoce na VFC na análise qualitativa de GR. Entretanto, não foram observadas diferenças estatísticas na análise quantitativa.

Takakura et al.,^21^ analisaram GR em pacientes que foram submetidos a transplante cardíaco, e observaram sinais quantitativos e qualitativos de recuperação de VFC, demonstrando que a reinervação autonômica cardíaca iniciou gradualmente após o transplante.

Acredita-se que o pequeno número de intervenções do Protocolo de Mobilização Precoce (apenas 4 intervenções) pode ter contribuído para a ausência de uma diferença estatisticamente significativa na análise quantitativa. É possível que um número mais alto de intervenções promova a melhoria da modulação autonômica em outros índices de VFC. Isso pode ser considerado um fator limitador deste estudo.

Para esta pesquisa, somente foram utilizados estudos que analisaram o GR após procedimentos cirúrgicos e/ou doenças, e não foram identificados estudos que analisaram os efeitos agudos do exercício físico nos pacientes com IMA ou Doença arterial coronariana (DAC). Isso sugere que são necessárias pesquisa adicionais para analisar a dinâmica de VFC não linear em relação a vários protocolos de mobilização precoce.

## Conclusão

O protocolo de mobilização precoce promove uma melhoria no comportamento autonômico, conforme avaliado por variabilidade de frequência cardíaca e por gráficos de recorrência, e pode ser considerado um procedimento útil para a melhor recuperação de pacientes submetidos a angioplastia coronária transluminal percutânea.
