# Supplementary material for: Influência de um Protocolo de Mobilização Precoce no comportamento autonômico de pacientes submetidos a Angioplastia Coronária Transluminal Percutânea
Source: Arq Bras Cardiol. 2021 Nov 22;117(6):1161–9. [Article in Portuguese] doi: 10.36660/abc.20200296 (PMC8757161; doi:10.36660/abc.20200296)
Supplement: Translation

# Translation: Influence of an Early Mobilization Protocol on the Autonomic Behavior of Patients Undergoing Percutaneous Transluminal Coronary Angioplasty

**Authors:** Bárbara Oliveira Silveira, Jade Lara de Melo, Graziella Paula de Oliveira Neri, Michele Lima Gregório, Moacir Fernandes de Godoy, Marilita Falangola Accioly

**Affiliations:** 1 Uberaba MG Brazil Universidade Federal do Triângulo Mineiro, Uberaba, MG – Brazil; 2 São Jose do Rio Preto SP Brazil Faculdade de Medicina de São José do Rio Preto (FAMERP) - Pós Graduação Enfermagem, São Jose do Rio Preto, SP – Brazil; 3 São Jose do Rio Preto SP Brazil Faculdade de Medicina de São José do Rio Preto (FAMERP) - Cardiologia e Cirurgia Cardiovascular, São Jose do Rio Preto, SP – Brazil

**Keywords:** Cardiovascular Diseases/mortality, Myocardial Infarction, Early Ambulation/methods, Exercise, Autonomic Nervous System, Angioplasty

## Abstract

**Background:**

Recurrence Plots (RP) enable a nonlinear analysis of Heart Rate Variability (HRV) and provide information on the Autonomic Nervous System (ANS).

**Objectives:**

To evaluate whether early ambulation in patients undergoing Percutaneous Transluminal Coronary Angioplasty (PTCA) influences the quantitative and qualitative components of RP.

**Methods:**

A total of 32 participants who underwent PTCA were divided into a Control Group (CG - no physical exercises) and an Early Ambulation Group (EAG – with physical exercises). Beat-to-beat heart rate was recorded using a heart rate monitor in both groups upon admission and discharge. The linear indices in the time and frequency domains were analyzed, and nonlinear indices were obtained through RP. The Early Ambulation Physical Therapy Protocol began 12-18 hours after PTCA. A two-tailed unpaired t-test was used for comparisons, and p-values < 0.05 were accepted as significant.

**Results:**

When comparing both groups, upon discharge, EAG showed an increase in SDNN (23.55 ± 12.05 to 37.29 ± 16.25; p=0.042), Triangular Index (8.99 ± 3.03 to 9.66 ± 3.07; p=0.014), and VLF (694.20 ± 468.20 to 848.37 ± 526.51; p=0.004), but without significant changes in the nonlinear evaluation. In addition, in the qualitative analysis of RP, a more diffuse and less geometric pattern was observed in EAG, indicating greater variability, while in CG, an altered and more geometric pattern was noted.

**Conclusion:**

The Early Ambulation Protocol promotes an improvement in autonomic behavior as evaluated by HRV and by RP, which can thus be considered a useful procedure for better recovery of patients undergoing PTCA.

## Introduction

Cardiovascular diseases account for the highest number of deaths worldwide.¹ Among them, Acute Myocardial Infarction (AMI) is the leading cause of death in Brazil.² However, survival of these patients has increased as a result of technological advances, such as Percutaneous Transluminal Coronary Angioplasty (PTCA).^3,4^

Associated with PTCA, multidisciplinary interaction plays an important role in reducing mortality,^5^ since early ambulation avoids bed confinement and its many deleterious effects, such as functional decline and reduced quality of life after discharge.^6^ However, it is still very common for patients to remain confined to a bed for fear of hemodynamic instability.^7^

In contrast, the analysis of cardiac autonomic modulation through Heart Rate Variability (HRV) has been used as a predictor of cardiovascular risks in various conditions.^8,9^ However, most studies used the linear analysis of HRV.^10-12^

The human body is a good illustration of a natural “Complex System” characterized by the continuous interaction of its multiple organs to maintain life. Its complexity results in a mode of behavior that is typically nonlinear in normal situations.^13^ Thus, the methods related to nonlinear dynamics are usually more clinically relevant for a better interpretation of the pathophysiological behavior of HRV under various conditions and, consequently, its prognostic value, complementing the information obtained with traditional evaluations.^14^

Recurrence Plots (RP) are a nonlinear analysis method idealized by Eckmann et al.,^15^ which proposes the analysis of the behavior of a system, represented by a time series, in an abstract space called phase space, enabling the quantification and qualification of HRV.^15,16^

In addition, little is known about acute responses to early exercise in autonomic modulation and cardiovascular function in the immediate postoperative period of patients undergoing myocardial revascularization and PTCA.^17^

Therefore, the present study aimed to evaluate whether early ambulation in patients undergoing PTCA influences the quantitative and qualitative components of RP.

## Methods

### Sample

This is a prospective, controlled and quasi-experimental clinical trial. The sample included individuals who underwent PTCA at the University Hospital of the University of Triângulo Mineiro.

The sample size was calculated considering the prevalence of individuals requiring angioplasty and hospitalized in a coronary care unit. For the calculation, the following formula was used: n= Z^2^ x p (p-100) / e^2^, where Z is the constant critical value that corresponds to the 95% confidence interval (95% CI); p is the prevalence of the disease/main variable; and e is sampling error, which may vary up to 10% of the true value of the population selected for the sample, suggesting a sample of 15 individuals for each group.

The flowchart for sample recruitment and selection is displayed in Figure 1.

**Figure 1:** Flowchart showing recruitment and selection of study participants. AMI: Acute Myocardial Infarction. CPA: Cardiopulmonary Arrest. PTCA: Percutaneous Transluminal Coronary Angioplasty. MV: Mechanical Ventilation.

The sample consisted of 32 participants who met the following inclusion criteria: be at least 18 years old; show a medical diagnosis of uncomplicated AMI (Killip I and II), with or without ST segment elevation and/or indication of elective PTCA (successful PTCA with residual stenosis of less than 50%); and be able to understand the instructions to perform the physical exercises. Next, the participants were divided into two groups: Early Ambulation (EAG) with 16 participants submitted to the Early Ambulation Protocol; and Control (GC) with 16 participants not submitted to the Early Ambulation Protocol. The sample was matched for age, sex, and medical diagnosis.

Individuals with at least one of the following characteristics were excluded from the study: history of previous AMI, complicated AMI (Killip III and IV), pacemaker implantation, 2^nd^ or 3^rd^ degree atrioventricular block, sequelae of stroke, inferior limb amputation, severe aortic stenosis, previous myocardial revascularization surgery, heart failure, hemodynamic instability at rest, worsening of the general clinical condition, feverish condition, and respiratory failure (need for mechanical ventilation).

The study procedures followed all the norms of CNS Resolution No. 466 and was approved by the Ethics and Research Committee of the Federal University of Triângulo Mineiro under Resolution 2.319.890 and the Brazilian Registry of Clinical Trials RBR-9w378x.

Figure 1 - Flowchart showing recruitment and selection of study participants. AMI: Acute Myocardial Infarction. CPA: Cardiopulmonary Arrest. PTCA: Percutaneous Transluminal Coronary Angioplasty. MV: Mechanical Ventilation.

### Experimental Protocol

The experimental protocol consisted of four phases. Phase I was conducted with interviews and assessments of medical records. Phase II was performed 12 to 18 hours after PTCA and consisted of recording beat-to-beat heart rate, using a Polar® heart rate monitor model RS800CX (Polar Kempele, Finland), and the tachogram was collected continuously for 20 minutes. During the entire collection process, the participant remained at rest, in the supine position, in silence and awake. Phase III was characterized by the implementation of an Early Ambulation Protocol, performed only by EAG.

In Phase IV, a new electrocardiogram was performed to analyze HRV, following the same procedures as in Phase II.

### Early Ambulation Protocol

The Early Ambulation Protocol was adapted from the protocol used at the Grady Memorial Hospital and Emory University School of Medicine,^18^ consisting of progressive steps in different positions, as described in Table 1. The protocol was started and applied in the positions according to the participants’ functional status, which was verified by evaluations and communication with the multidisciplinary team. The EAG performed the protocol throughout the hospitalization period, which consisted of two sessions per day (4 interventions during ICU stay).

**Table 1:** Early Ambulation Protocol

Position	Exercises
Supine	A) One-set diaphragmatic exercises associated with active-assisted or active-free diagonal movement of the upper limb (10 times for each limb);
	B) Repeat the exercise over 1 set with both members simultaneously (10 times);
	C) Repeat the exercise over 1 set with both members simultaneously (10 times);
	D) Active assisted or active free lower limbs - triple flexion (10 times) + abduction / hip adduction (10 times for each limb);
	E) Ankle flexion-extension (10 times) for each foot;
	F) Ankle circumference (10 times) for each foot.
Sitting	Active exercises from A to F
Orthostatism	Active exercises from A to F and active for lower limbs (supported if necessary)
	G) Standing on tiptoe (10 times) with both limbs simultaneously;
	H) Half squats (10 times);
	I) Stationary gait (for 30 seconds);
	J) Ambulation in the room (1 round).

The following criteria were used to discontinue the Early Ambulation Protocol: signs and/or symptoms of fatigue, chest pain, dyspnea, cyanosis, pallor, tachycardia (>120 beats per minute), bradycardia, complex arrhythmias, and hypotension (Mean Blood Pressure <65mmHg).

### Evaluation of autonomic modulation

For the analysis of HRV indices, RR interval records were transmitted to a computer using the Polar Precision Performance software (version 4.01.029)^19^ and converted to text files. Only those series with more than 95% of sinus beats were analyzed, following the selection of the 1,000 most stable points (Kubios HRV Software, version 2.0, University of Kuopio, Finland). The data were filtered using the Polar Precision Performance standard software filter (version 4.01.029), with a moderate filter. Next, a graphical computational filtering tool called T-RR Filter^20^ was used.

Among the linear methods, the time domain measures normal RR intervals (iR-R), and various measurements are calculated from these intervals, including: standard deviation of the mean normal iR-R (SDNN), which corresponds to sympathetic and parasympathetic effects and represents global variability; the percentage of adjacent iR-Rs with a difference of duration greater than 50 milliseconds (pNN50); and the root mean square of the successive differences between the usual adjacent iR-Rs (RMSSD). The RMSSD and pNN50 variables are related only to parasympathetic behavior, while SDNN reflects all components responsible for variability. The Triangular Index refers to the number of all iR-Rs divided by the frequency of these iR-Rs in the modal compartment of the histogram, thus reflecting the global variability.^21^

For the analysis of the frequency domain, the cubic splines interpolation method at 4Hz was applied and the power spectral density of the most stable segment was calculated using the Fast Fourier Transform (FFT), calculating, in milliseconds squared (ms^2^), the spectral components for Very Low Frequency (VLF) (<0.04Hz), Low Frequency (LF) (0.04-0.15 Hz), and High Frequency (HF) (0.15 to 0.40 Hz), in addition to the ratio of these components (LF/HF).

LF represents the sympathetic and parasympathetic modulation; HF represents the vagal modulation activity.^22^ VLF reflects humoral, vascular, thermal regulations, as well as the activity of the renin-angiotensin-aldosterone system.^22^ Moreover, the LF and HF ratio can be considered a measure of sympathovagal balance.^22,23^

RP were used for nonlinear analysis of HRV, which was made in a qualitative and quantitative manner. The qualitative analysis was performed by visualizing the graphic pattern and the quantitative analysis used the following indices: Recurrence (REC), Determinism (DET), Shannon entropy (ES), Laminarity (LAM), Trapping time (TT), and Maximum line (MaxLine).^15^ The following parameters were used in RP: embedding dimension = 10; time delay = 1, radius = 70, line = 2,^24^ and color scheme = Volcano.

A time series is used for the construction of RP. According to the ranges of values between measurements (dimension) and distances or time intervals (radius), it can be verified whether or not recurrence values exist. The use of different colors represents different radii complementing the typical visual appearance of RP.^24^

RP can be analyzed by small and large scale patterns. The small scale patterns fit the points, diagonal and vertical lines, which enable a qualitative analysis. For example, in healthy individuals, RP show a diagonal line and fewer apparent squares, indicating higher HRV. In individuals with some autonomic modulation impairment, RP show more squares defined in the graph, more geometric shapes, indicating the inherent periodicity, linear behavior and a low HRV.^24,25^

In the quantitative analysis, some indices are generated: recurrence rate (REC%), which quantifies the percentage of recurrent points within a specific radius; Percentage of Determinism (DET%), representing the diagonals formed by recurrence points; average Length of Diagonal Lines (Lmean) and Maximum Length of Diagonal Lines (Lmax), representing the largest diagonal except the main one; Laminarity (LAM), which are the recurrence points that form vertical lines; Trapping Time (TT), which is the average length of vertical lines; Entropy, representing Shannon's entropy, which measures the distribution complexity of diagonal lines. In this case, unlike other interpretations, it is understood that the higher the Shannon entropy is, the more linear the time series will be.^25^

For the qualitative analysis, chaotic, randomized, periodic, and linear systems constructed by mathematical formulation reported by Takakura et al.^24^ were used as RP models, as shown in Figure 2.

**Figure 2:** RP of randomized, chaotic, periodic, and linear systems, obtained with mathematical formulation.

Source: Takakura et al.^21^

### Statistical Analysis

A descriptive analysis of continuous and categorical variables was performed. The presence or absence of normal distribution of variables was assessed by Shapiro-Wilk tests. Continuous data with Gaussian distribution were expressed as mean ± standard deviation. Categorical variables were expressed as absolute and percentage values.

A two-tailed unpaired t-test was set for all intergroup analyses being admitted an alpha error of 5%. All analyses were performed with Stats Direct Statistical Software version 3.3.3.

## Results

The analyzed sample was homogeneous regarding clinical diagnosis, systemic blood pressure, heart rate at rest, length of hospital stay, number of implanted stents, and HRV indices. Table 2 describes the sample characterization of both groups.

**Table 2:** Sample Characterization

	Control Group (N=16)	Early Ambulation Group (N=16)	p-values*
**Sociodemographic profile**		
Age: years	61.00 ±8.34	63.50 ±9.64	0.439
Sex: male (n)	08 (50%)	08 (50%)	
BMI (kg/m^2^)	27 ±3	29 ±3	0.467
**Diagnostic**			
Coronary Artery Disease (n)	08 (50%)	08 (50%)	
Acute Myocardial Infarction (n)	08 (50%)	08 (50%)	
**BP / HR (at rest)**			
Heart Rate (bpm)	71.38 ±11.09	71.19 ±10.08	0.960
Systolic blood pressure (mmHg)	130.25 ±22.62	134.38±19.26	0.583
Diastolic Blood Pressure (mmHg)	76.50 ±17.29	72.06 ±9.95	0.383
Mean blood pressure (mmHg)	94.06 ±21.22	92.69 ±13.05	0.827
**Coronary obstruction**			
Right Coronary Artery (n)	12 (75%)	10 (62.5%)	
Anterior Descending (n)	8 (50%)	8 (50%)	
Circumflex (n)	2 (12.5%)	3 (18.8%)	
**Length of stay in hospital (days)**	3.94 ±1.48	3.75 ±1.18	0.113
**Number of stents**	1.63 ±0.71	1.94 ±0.85	0.215

N: number of participants; mean ± standard deviation; BM: body mass index; kg/m2; kilogram per square meter; BP: blood pressure; HR: heart rate; bpm: beats per minute; mmHg: millimeters of mercury.

* unpaired Student's t-test.

The drugs used by CG were beta-blockers (81.3%), hypolipidemics (62.5%), Ieca (50%), Aspirin (62.5%), and Ticlopidine/clopidogrel (18.8), while EAG used beta-blockers (87.5%), hypolipidemics (53.6%), Ieca (50%), Aspirin (62.5%), and Ticlopidine/clopidogrel (37.5%).

Table 3 summarizes the analysis of linear indices for the time and frequency domains, in which we can observe a statistically significant difference in SDNN, Triangular Index, and VLF when comparing CG with EAG. Table 4 shows the quantitative analysis of nonlinear indices by the Visual Recurrence Analysis Software. There was no statistical difference between both moments when comparing CG and EAG.

**Table 3:** Heart Rate Variability analyzed by Linear Methods: Control vs. Early Ambulation

HRV indices	Control		Early Ambulation		Control x Early Ambulation	
	Admission	Discharge	Admission	Discharge	Admission	Discharge
	Mean (SD)	Mean (SD)	Mean (SD)	Mean (DP)	p-value	p-value
**Time Domain**						
Mean RR	894.58 ±112.65	825.29 ±85.04	890.60 ±147.14	908.12 ±140.25	0.9321	0.0544
SDNN	27.73 ±18.91	27.4 ±8.71	23.55 ±12.05	37.29 ±16.25	0.4610	0.0428*
RMSSD	21.98 ±11.10	15.69 ±9.09	19.68 ±11.03	20.48 ±13.47	0.5601	0.2482
PNN50	4.19 ±7.65	3.83 ±6.85	2.30 ±5.26	5.12 ±6.53	0.7046	0.4864
Triangular Index	9.34 ±4.99	7.23 ±2.09	8.99 ±3.03	9.66 ±3.07	0.8126	0.0148*
TINN	142.81 ±54.25	131.25 ±39.39	120.62 ±63.03	130.0 ±70.75	0.2944	0.9513
**Frequency Domain**						
VLF power (ms^2^)	927.23 ±1395.20	407.56 ±237.61	694.20 ±468.20	848.37 ±526.51	0.5308	0.0043*
LF power (ms^2^)	300.06 ±367.49	177.81 ±154.94	233.93 ±165.23	380.50 ±513.86	0.5165	0.1414
HF power (ms^2^)	148.43 ±174.09	159.87 ±223.07	94.12 ±117.58	177.68 ±159.76	0.4677	0.3282
LF/HF power (ms^2^)	2.86 ±2.12	3.39 ±2.49	2.80 ±1.61	3.94 ±3.65	0.9295	0.6205

Mean RR: mean of RR intervals; RMSSD: root mean square of the successive differences between the usual adjacent iR-Rs; pNN50: percentage of adjacent RR intervals that differ by duration greater than 50 milliseconds; SDNN: standard deviation of the average of normal RR intervals; RR tri index: triangular index RR; TINN: Triangular interpolation of the NN interval histogram. Data expressed as mean (standard deviation); VLF: Very Low Frequency; HF: High Frequency; LF: Low Frequency; LF/HF: Ratio between Low and High Frequency

* statistically significant difference (p <0.05). unpaired Student's t-test (Control x Early Ambulation).

**Table 4:** Heart Rate Variability analyzed by Nonlinear Methods: Control x Early Ambulation

HRV indices	Control		Early Ambulation		Control x Early Ambulation	
	Admission	Discharge	Admission	Discharge	Admission	Discharge
	Mean (SD)	Mean (SD)	Mean (SD)	Mean (SD)	p-value	p-value
Visual Recurrence Analysis (VRA)						
Mean	890.64±147.15	908.22±140.36	893.71±101.13	843.18±90.72	0.9457	0.1318
Percentage of Recurrence	40.71 ±7.56	40.18 ±2.41	38.54 ±3.15	43.60±13.66	0.3021	0.3389
Percentage of Determinism	86.35 ±10.99	86.65 ±11.37	83.71 ±13.78	86.63±11.39	0.553	0.8099
Percentage of Laminarity	92.38 ±6.29	91.05 ±9.61	89.62 ±9.66	91.51 ±9.77	0.3477	0.8941
Trapping Time	11.65 ±12.15	8.73 ±3.11	7.68 ±3.67	7.82 ±2.40	0.2271	0.3668
Ratio	2.14 ±0.23	2.12 ±0.22	2.16 ±0.25	2.14 ±0.28	0.8472	0.8257
Entropy	3.79 ±0.75	3.83 ±0.52	3.66 ±0.60	3.71 ±0.48	0.5886	0.5117
MaxLine	219.93±191.23	241.68±223.29	238.12±166.34	171.12±95.56	0.7761	0.2587

Data expressed as mean (standard deviation) *: statistically significant difference (p <0.05). Paired t-test (parametric samples) and Wilcoxon test (nonparametric sample). unpaired Student's t-test (Control x Early Ambulation).

However, an overall worsening of the Control Group pattern can be observed in Figure 3, which shows the visual (qualitative) analysis of RP. Upon discharge, there are a greater number of dark squares and more geometric shapes, indicating higher linearity, and therefore less system homeostasis when compared with admission. Moreover, when comparing EAG with GC, a more chaotic pattern can be observed, with fewer apparent and more homogeneous squares in EAG, indicating greater complexity and improvement of homeostasis.

**Figure 3:** Recurrence Plots of participants identified by P_00 admission and discharge.

Source: Research

## Discussion

The major findings of the intragroup analysis of EAG were the significant increase in the linear indices (SDNN and PNN50) upon discharge, when compared with those upon admission. In the EAG intergroup analysis, a chaotic and more complex pattern was observed.

This increase in linear indices is indicative of high HRV, represented by the increase in parasympathetic activity in PNN50 and global autonomic activity in SDNN, following the application of the Early Ambulation Protocol. This result infers improvement of overall health of the cardiovascular system, given that increased parasympathetic activity is related to higher HRV, and lower cardiovascular mortality,^26,27^ demonstrating the beneficial influence of exercises performed in the ICU.

In the intergroup analysis, the indices for SDNN, Triangular Index, and VLF show a statistically significant difference upon discharge. The SDNN indices infer the global information on both parasympathetic and sympathetic modulation; the Triangular Index expresses RR interval variability, closely related to SDNN; and VLF is related to the angiotensin-aldosterone renin system, thermoregulation, and peripheral vasomotor tone.^25^

Some studies^27,28^ have presented evidence that early ambulation improves autonomic modulation in individuals after AMI. However, these studies used linear methods for their analyses, and recent studies point out that our body has a nonlinear behavior. Therefore, the analysis of the autonomic modulation by nonlinear methods is paramount.^14^

Nonlinear analysis, unlike linear analysis, measures quality, scale, and correlation of signal properties, thus providing an interpretation of the unpredictability, complexity, and fractability of the system.^14^ Meyerfeldt et al.^26^ found higher “sensitivity” of nonlinear analysis when compared with linear analysis in pathological behaviors, such as tachyarrhythmias.

When analyzing the results of indices for chaos domains, in absolute values, we found no statistically significant differences. However, when analyzing the qualitative aspect of RP (Figure 3), it is possible to notice that the RP of the participants treated with early ambulation show improvement in HRV, as they present more heterogeneous colors and less geometric patterns. When compared with the mathematical models (Figure 2), they show a behavior that tends to chaos, indicating greater variability, and therefore autonomic improvement. The study conducted by Manata et al.^30^ supports our findings, which used RP to compare healthy individuals with individuals with Chronic Obstructive Pulmonary Disease (COPD). The authors concluded that individuals with COPD had lower HRV, as the RP showed more visible squares and a more homogeneous configuration. They further observed higher recurrence points when compared with healthy individuals, which indicates a more recurrent and less dynamic system and less complex autonomic modulation in this population.

Godoy & Gregório,^31^ when analyzing the RP of several groups, were able to verify a difference in the variability of these individuals (newborns, adults, renal patients, and individuals with declared brain death), for the quantitative and qualitative analyses of RP. This evidence seems to corroborate our findings, as we observed a positive influence of early ambulation on HRV for the qualitative analysis of RP. However, we did not observe statistical differences in the quantitative analysis.

Takakura et al.^21^ analyzed RP in patients who underwent heart transplantation and observed quantitative and qualitative signs of HRV recovery, showing that the heart autonomic re-innervation started gradually after transplantation.

We believe that the small number of interventions of the Early Ambulation Protocol (only 4 interventions) may have contributed to the absence of a statistically significant difference in the quantitative analysis. A larger number of interventions are likely to promote improvement of autonomic modulation in other HRV indices. This can be considered a limiting factor of our study.

This research only used studies that analyzed RP after surgical procedures and/or conditions, and no studies were found that analyzed the acute effects of physical exercise on patients with AMI or Coronary Artery Disease (CAD). This suggests that further research is required to analyze the nonlinear HRV dynamics in relation to different early ambulation protocols.

## Conclusion

The Early Ambulation Protocol promotes an improvement in autonomic behavior as evaluated by Heart Rate Variability as well as by Recurrence Graphs, which can then be considered a useful procedure for a better recovery of patients undergoing percutaneous transluminal coronary angioplasty.
